# Supplementary material for: Native Rhizobial Inoculation Improves Tomato Yield and Nutrient Uptake While Mitigating Heavy Metal Accumulation in a Conventional Farming System
Source: Microorganisms. 2025 Aug 15;13(8):1904. doi: 10.3390/microorganisms13081904 (PMC12388614; doi:10.3390/microorganisms13081904)
Supplement: Supplementary file 1 [file microorganisms-13-01904-s001.zip › FigS1.pdf]

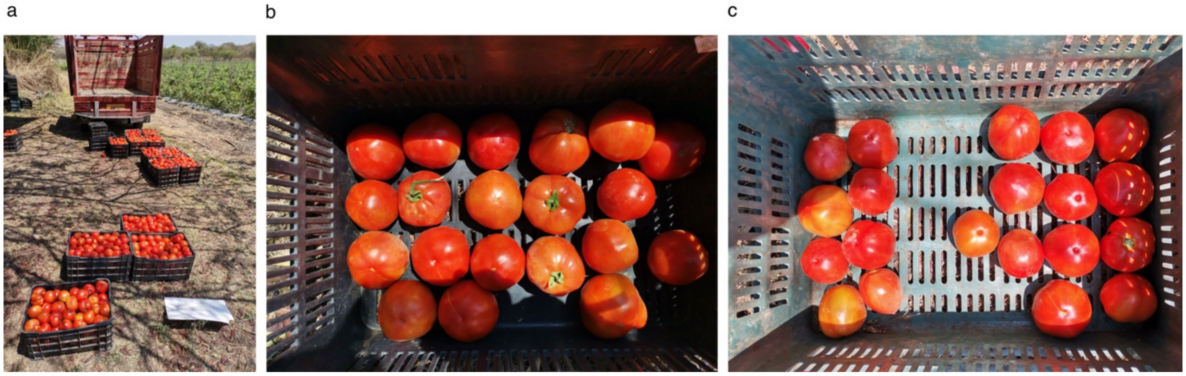

Fig. S1 Effect of native rhizobial bacteria inoculation on tomato cultivation. (a) Overview of harvested tomatoes, (b) Tomatoes from plants inoculated with Rhizobium sp. ACO-34A strain, (c) Tomatoes from non-inoculated plants.
