# Supplementary material for: Native Rhizobial Inoculation Improves Tomato Yield and Nutrient Uptake While Mitigating Heavy Metal Accumulation in a Conventional Farming System
Source: Microorganisms. 2025 Aug 15;13(8):1904. doi: 10.3390/microorganisms13081904 (PMC12388614; doi:10.3390/microorganisms13081904)
Supplement: Supplementary file 1 [file microorganisms-13-01904-s001.zip › Table S1.pdf]

Supplementary Table 1. Pearson correlation coefficients (r) and p-values between nutrient content accumulation in plant tissue, morphometric characteristics, and fruit yield parameters in tomato plants.

Pearson correlation (*r*)

Parameters	N (g plant^{-1})	P (g plant^{-1})	K (g plant^{-1})	Ca (g plant^{-1})	Mg (g plant^{-1})	S (g plant^{-1})	Na (mg plant^{-1})	Fe (mg plant^{-1})	Zn (mg plant^{-1})	Mn (mg plant^{-1})	Cu (mg plant^{-1})	B (mg plant^{-1})	Ni (mg plant^{-1})	Mo (mg plant^{-1})	Plant height (cm)	Plant stem width (mm)	Plant dry weight (g)	Chlorophyll (SPAD index)	Fruits plant^{-1}	Fruit height (cm)	Fruit width (mm)	EFV (cm^{3})	Fruit weight (g)
N (g plant^{-1})	1.0000	0.9621	0.9598	0.9649	0.7663	0.9402	0.8728	0.3627	0.9348	0.8251	0.7301	0.9135	0.4954	0.5846	0.6142	0.1039	0.9877	-0.0104	0.1059	-0.2791	-0.3919	-0.4058	-0.4027
P (g plant^{-1})	0.9621	1.0000	0.9562	0.9787	0.7679	0.9316	0.9287	0.1926	0.9791	0.7576	0.6274	0.8484	0.3360	0.3901	0.5871	0.2457	0.9730	0.0454	0.1516	-0.0655	-0.2266	-0.2222	-0.2092
K (g plant^{-1})	0.9598	0.9562	1.0000	0.9090	0.8806	0.8359	0.9649	0.1049	0.9171	0.6381	0.5149	0.7676	0.2458	0.3722	0.4935	0.2988	0.9525	0.1496	0.2685	-0.1758	-0.1745	-0.2115	-0.2350
Ca (g plant^{-1})	0.9649	0.9787	0.9090	1.0000	0.6676	0.9828	0.8577	0.3317	0.9749	0.8547	0.7402	0.9171	0.4691	0.4976	0.6905	0.1467	0.9728	-0.0527	0.0259	-0.1362	-0.3755	-0.3569	-0.3287
Mg (g plant^{-1})	0.7663	0.7679	0.8806	0.6676	1.0000	0.5475	0.8917	-0.0720	0.7640	0.3511	0.2676	0.5170	0.0385	0.1146	0.1150	0.2299	0.7976	0.0312	0.3465	-0.0044	0.0594	0.0370	0.0191
S (g plant^{-1})	0.9402	0.9316	0.8359	0.9828	0.5475	1.0000	0.7514	0.4763	0.9291	0.9293	0.8359	0.9614	0.6019	0.6150	0.7403	0.0223	0.9402	-0.1136	-0.0515	-0.2002	-0.4844	-0.4566	-0.4213
Na (mg plant^{-1})	0.8728	0.9287	0.9649	0.8577	0.8917	0.7514	1.0000	-0.1221	0.9060	0.4872	0.3357	0.6286	0.0229	0.1252	0.4186	0.4593	0.8881	0.2071	0.3337	0.0175	0.0214	-0.0073	-0.0280
Fe (mg plant^{-1})	0.3627	0.1926	0.1049	0.3317	-0.0720	0.4763	-0.1221	1.0000	0.2220	0.7642	0.8771	0.6768	0.9885	0.8951	0.3702	-0.7738	0.3445	-0.5871	-0.4699	-0.4760	-0.8192	-0.7526	-0.6820
Zn (mg plant^{-1})	0.9348	0.9791	0.9171	0.9749	0.7640	0.9291	0.9060	0.2220	1.0000	0.7726	0.6551	0.8547	0.3598	0.3460	0.5786	0.1731	0.9717	-0.0996	0.0660	0.0097	-0.2510	-0.2147	-0.1792
Mn (mg plant^{-1})	0.8251	0.7576	0.6381	0.8547	0.3511	0.9293	0.4872	0.7642	0.7726	1.0000	0.9781	0.9817	0.8503	0.8059	0.6979	-0.3115	0.8225	-0.3450	-0.2537	-0.3176	-0.6931	-0.6375	-0.5761
Cu (mg plant^{-1})	0.7301	0.6274	0.5149	0.7402	0.2676	0.8359	0.3357	0.8771	0.6551	0.9781	1.0000	0.9415	0.9373	0.8642	0.6128	-0.4844	0.7288	-0.4709	-0.3396	-0.3674	-0.7646	-0.6974	-0.6257
B (mg plant^{-1})	0.9135	0.8484	0.7676	0.9171	0.5170	0.9614	0.6286	0.6768	0.8547	0.9817	0.9415	1.0000	0.7793	0.7690	0.6769	-0.2174	0.9112	-0.2730	-0.1518	-0.3151	-0.6284	-0.5894	-0.5447
Ni (mg plant^{-1})	0.4954	0.3360	0.2458	0.4691	0.0385	0.6019	0.0229	0.9885	0.3598	0.8503	0.9373	0.7793	1.0000	0.9232	0.4595	-0.6959	0.4770	-0.5453	-0.4315	-0.4784	-0.8289	-0.7670	-0.6995
Mo (mg plant^{-1})	0.5846	0.3901	0.3722	0.4976	0.1146	0.6150	0.1252	0.8951	0.3460	0.8059	0.8642	0.7690	0.9232	1.0000	0.4985	-0.5205	0.5137	-0.2521	-0.2190	-0.6648	-0.7827	-0.7923	-0.7784
Plant height (cm)	0.6142	0.5871	0.4935	0.6905	0.1150	0.7403	0.4186	0.3702	0.5786	0.6979	0.6128	0.6769	0.4595	0.4985	1.0000	0.1332	0.5758	0.1069	-0.2673	-0.5062	-0.5758	-0.6273	-0.6348
Plant stem width (mm)	0.1039	0.2457	0.2988	0.1467	0.2299	0.0223	0.4593	-0.7738	0.1731	-0.3115	-0.4844	-0.2174	-0.6959	-0.5205	0.1332	1.0000	0.0768	0.6465	0.3331	0.1424	0.4444	0.3327	0.2643
Plant dry weight (g)	0.9877	0.9730	0.9525	0.9728	0.7976	0.9402	0.8881	0.3445	0.9717	0.8225	0.7288	0.9112	0.4770	0.5137	0.5758	0.0768	1.0000	-0.0948	0.0820	-0.1679	-0.3529	-0.3407	-0.3211
Chlorophyll (SPAD index)	-0.0104	0.0454	0.1496	-0.0527	0.0312	-0.1136	0.2071	-0.5871	-0.0996	-0.3450	-0.4709	-0.2730	-0.5453	-0.2521	0.1069	0.6465	-0.0948	1.0000	0.6461	-0.0970	0.4768	0.3109	0.1318
Fruits plant^{-1}	0.1059	0.1516	0.2685	0.0259	0.3465	-0.0515	0.3337	-0.4699	0.0660	-0.2537	-0.3396	-0.1518	-0.4315	-0.2190	-0.2673	0.3331	0.0820	0.6461	1.0000	0.2422	0.7227	0.6447	0.5250
Fruit height (cm)	-0.2791	-0.0655	-0.1758	-0.1362	-0.0044	-0.2002	0.0175	-0.4760	0.0097	-0.3176	-0.3674	-0.3151	-0.4784	-0.6648	-0.5062	0.1424	-0.1679	-0.0970	0.2422	1.0000	0.6553	0.8034	0.8629
Fruit width (mm)	-0.3919	-0.2266	-0.1745	-0.3755	0.0594	-0.4844	0.0214	-0.8192	-0.2510	-0.6931	-0.7646	-0.6284	-0.8289	-0.7827	-0.5758	0.4444	-0.3529	0.4768	0.7227	0.6553	1.0000	0.9724	0.9142
EFV (cm^{3})	-0.4058	-0.2222	-0.2115	-0.3569	0.0370	-0.4566	-0.0073	-0.7526	-0.2147	-0.6375	-0.6974	-0.5894	-0.7670	-0.7923	-0.6273	0.3327	-0.3407	0.3109	0.6447	0.8034	0.9724	1.0000	0.9762
Fruit weight (g)	-0.4027	-0.2092	-0.2350	-0.3287	0.0191	-0.4213	-0.0280	-0.6820	-0.1792	-0.5761	-0.6257	-0.5447	-0.6995	-0.7784	-0.6348	0.2643	-0.3211	0.1318	0.5250	0.8629	0.9142	0.9762	1.0000

correlation *p*-values

	N (g plant^{-1})	P (g plant^{-1})	K (g plant^{-1})	Ca (g plant^{-1})	Mg (g plant^{-1})	S (g plant^{-1})	Na (mg plant^{-1})	Fe (mg plant^{-1})	Zn (mg plant^{-1})	Mn (mg plant^{-1})	Cu (mg plant^{-1})	B (mg plant^{-1})	Ni (mg plant^{-1})	Mo (mg plant^{-1})	Plant height (cm)	Plant stem width (mm)	Plant dry weight (g)	Chlorophyll (SPAD index)	Fruits plant^{-1}	Fruit height (cm)	Fruit width (mm)	EFV (cm^{3})	Fruit weight (g)
N (g plant^{-1})	0.0000	0.0000	0.0000	0.0000	0.0037	0.0000	0.0002	0.2466	0.0000	0.0010	0.0070	0.0000	0.1015	0.0459	0.0336	0.7480	0.0000	0.9744	0.7431	0.3797	0.2077	0.1905	0.1944
P (g plant^{-1})	0.0000	0.0000	0.0000	0.0000	0.0035	0.0000	0.0000	0.5487	0.0000	0.0043	0.0290	0.0005	0.2856	0.2099	0.0448	0.4414	0.0000	0.8886	0.6382	0.8398	0.4788	0.4875	0.5141
K (g plant^{-1})	0.0000	0.0000	0.0000	0.0000	0.0002	0.0007	0.0000	0.7456	0.0000	0.0256	0.0867	0.0036	0.4413	0.2334	0.1030	0.3455	0.0000	0.6425	0.3988	0.5847	0.5876	0.5093	0.4621
Ca (g plant^{-1})	0.0000	0.0000	0.0000	0.0000	0.0177	0.0000	0.0004	0.2922	0.0000	0.0004	0.0059	0.0000	0.1239	0.0998	0.0129	0.6491	0.0000	0.8708	0.9362	0.6729	0.2290	0.2548	0.2968
Mg (g plant^{-1})	0.0037	0.0035	0.0002	0.0177	0.0000	0.0654	0.0001	0.8241	0.0038	0.2631	0.4005	0.0852	0.9054	0.7229	0.7219	0.4723	0.0019	0.9234	0.2699	0.9891	0.8546	0.9092	0.9531
S (g plant^{-1})	0.0000	0.0000	0.0007	0.0000	0.0654	0.0000	0.0048	0.1175	0.0000	0.0000	0.0007	0.0000	0.0384	0.0333	0.0059	0.9451	0.0000	0.7252	0.8738	0.5326	0.1105	0.1357	0.1726
Na (mg plant^{-1})	0.0002	0.0000	0.0000	0.0004	0.0001	0.0048	0.0000	0.7055	0.0000	0.1082	0.2861	0.0286	0.9438	0.6983	0.1757	0.1331	0.0001	0.5185	0.2891	0.9569	0.9473	0.9820	0.9311
Fe (mg plant^{-1})	0.2466	0.5487	0.7456	0.2922	0.8241	0.1175	0.7055	0.0000	0.4881	0.0038	0.0002	0.0156	0.0000	0.0001	0.2362	0.0031	0.2728	0.0448	0.1233	0.1177	0.0011	0.0047	0.0146
Zn (mg plant^{-1})	0.0000	0.0000	0.0000	0.0000	0.0038	0.0000	0.0000	0.4881	0.0000	0.0032	0.0208	0.0004	0.2507	0.2706	0.0487	0.5905	0.0000	0.7582	0.8384	0.9762	0.4313	0.5028	0.5772
Mn (mg plant^{-1})	0.0010	0.0043	0.0256	0.0004	0.2631	0.0000	0.1082	0.0038	0.0032	0.0000	0.0000	0.0000	0.0005	0.0016	0.0116	0.3243	0.0010	0.2721	0.4262	0.3145	0.0124	0.0257	0.0499
Cu (mg plant^{-1})	0.0070	0.0290	0.0867	0.0059	0.4005	0.0007	0.2861	0.0002	0.0208	0.0000	0.0000	0.0000	0.0000	0.0003	0.0341	0.1105	0.0072	0.1223	0.2801	0.2401	0.0038	0.0117	0.0295
B (mg plant^{-1})	0.0000	0.0005	0.0036	0.0000	0.0852	0.0000	0.0286	0.0156	0.0004	0.0000	0.0000	0.0000	0.0028	0.0035	0.0156	0.4972	0.0000	0.3906	0.6377	0.3184	0.0286	0.0437	0.0671
Ni (mg plant^{-1})	0.1015	0.2856	0.4413	0.1239	0.9054	0.0384	0.9438	0.0000	0.2507	0.0005	0.0000	0.0028	0.0000	0.0000	0.1329	0.0120	0.1169	0.0667	0.1614	0.1156	0.0009	0.0036	0.0113
Mo (mg plant^{-1})	0.0459	0.2099	0.2334	0.0998	0.7229	0.0333	0.6983	0.0001	0.2706	0.0016	0.0003	0.0035	0.0000	0.0000	0.0990	0.0828	0.0876	0.4293	0.4941	0.0183	0.0026	0.0021	0.0029

Plant height (cm)	0.0336	0.0448	0.1030	0.0129	0.7219	0.0059	0.1757	0.2362	0.0487	0.0116	0.0341	0.0156	0.1329	0.0990	0.0000	0.6799	0.0501	0.7408	0.4009	0.0931	0.0501	0.0290	0.0266
Plant stem width (mm)	0.7480	0.4414	0.3455	0.6491	0.4723	0.9451	0.1331	0.0031	0.5905	0.3243	0.1105	0.4972	0.0120	0.0828	0.6799	0.0000	0.8126	0.0231	0.2900	0.6588	0.1478	0.2907	0.4065
Plant dry weight (g)	0.0000	0.0000	0.0000	0.0000	0.0019	0.0000	0.0001	0.2728	0.0000	0.0010	0.0072	0.0000	0.1169	0.0876	0.0501	0.8126	0.0000	0.7695	0.8001	0.6020	0.2605	0.2785	0.3088
Chlorophyll (SPAD index)	0.9744	0.8886	0.6425	0.8708	0.9234	0.7252	0.5185	0.0448	0.7582	0.2721	0.1223	0.3906	0.0667	0.4293	0.7408	0.0231	0.7695	0.0000	0.0232	0.7644	0.1170	0.3254	0.6831
Fruits plant^{-1}	0.7431	0.6382	0.3988	0.9362	0.2699	0.8738	0.2891	0.1233	0.8384	0.4262	0.2801	0.6377	0.1614	0.4941	0.4009	0.2900	0.8001	0.0232	0.0000	0.4482	0.0079	0.0236	0.0797
Fruit height (cm)	0.3797	0.8398	0.5847	0.6729	0.9891	0.5326	0.9569	0.1177	0.9762	0.3145	0.2401	0.3184	0.1156	0.0183	0.0931	0.6588	0.6020	0.7644	0.4482	0.0000	0.0207	0.0016	0.0003
Fruit width (mm)	0.2077	0.4788	0.5876	0.2290	0.8546	0.1105	0.9473	0.0011	0.4313	0.0124	0.0038	0.0286	0.0009	0.0026	0.0501	0.1478	0.2605	0.1170	0.0079	0.0207	0.0000	0.0000	0.0000
EFV* (cm^3)	0.1905	0.4875	0.5093	0.2548	0.9092	0.1357	0.9820	0.0047	0.5028	0.0257	0.0117	0.0437	0.0036	0.0021	0.0290	0.2907	0.2785	0.3254	0.0236	0.0016	0.0000	0.0000	0.0000
Fruit weight (g)	0.1944	0.5141	0.4621	0.2968	0.9531	0.1726	0.9311	0.0146	0.5772	0.0499	0.0295	0.0671	0.0113	0.0029	0.0266	0.4065	0.3088	0.6831	0.0797	0.0003	0.0000	0.0000	0.0000

*EFV, Estimated Fruit Volume
